# Ecology, invasion history and biodiversity-driven management of the coconut black-headed caterpillar *Opisina arenosella* in Asia

**DOI:** 10.3389/fpls.2023.1116221

**Published:** 2023-03-27

**Authors:** Hui Lu, Baoqian Lyu, Jihong Tang, Qiqi Wu, Kris A. G. Wyckhuys, Khac Hoang Le, Patchareewan Chongchitmate, Haiyan Qiu, Qikai Zhang

**Affiliations:** ^1^ Environment and Plant Protection Institute, Chinese Academy of Tropical Agricultural Sciences, Key Laboratory of Integrated Pest Management on Tropical Crops, Ministry of Agriculture and Rural Affairs, Haikou, China; ^2^ Sanya Research Academy, Chinese Academy of Tropical Agriculture Science, Hainan Key Laboratory for Biosafety Monitoring and Molecular Breeding in Off-Season Reproduction Regions, Sanya, Hainan, China; ^3^ School of Biological Sciences, University of Queensland, St Lucia, QLD, Australia; ^4^ Institute for Plant Protection, Chinese Academy of Agricultural Sciences (CAAS), Beijing, China; ^5^ Chrysalis Consulting, Danang, Vietnam; ^6^ Plant Protection Department, Agronomy Faculty, Nong Lam University, Hochiminh City, Vietnam; ^7^ Plant Protection Research and Development Office, Department of Agriculture, Kasetsart University, Bangkok, Thailand

**Keywords:** coconut black-headed caterpillar, invasion biology, agroecology, crop protection, natural enemies, biological control, ecological intensification

## Abstract

The coconut black-headed caterpillar (BHC), *Opisina arenosella* Walker (Lepidoptera: Xyloryctidae) is an important herbivore of palm trees that originates in South Asia. Over the past decades, *O. arenosella* has spread to several countries in Eastern and Southeast Asia. BHC larval feeding can cause severe defoliation and occasional plant death, resulting in direct production losses (e.g., for coconut) while degrading the aesthetic value of urban and rural landscapes. In this review paper, we systematically cover taxonomy, bio-ecology, invasion history and current management of *O. arenosella* throughout Asia. Given that *O. arenosella* is routinely controlled with insecticides, we equally explore options for more sustainable management through agroecological and biodiversity-based tactics e.g., cultural control or biological control. Also, recent advances in chemical ecology have unlocked lucrative opportunities for volatile-mediated monitoring, mating disruption and mass-trapping. Substantial progress has been made in augmentation biological control, with scheduled releases of laboratory-reared parasitoids lowering BHC infestation pressure up to 95%. Equally, resident ants provide 75-98% mortality of BHC egg masses within the palm canopy. Biological control has been effectively paired with sanitary measures and good agronomy (i.e., proper fertilization, irrigation), and promoted through participatory farmer training programs. Our comprehensive listing of non-chemical preventative and curative tactics offer bright prospects for a more environmentally-sound, biodiversity-driven mitigation of a palm pest of regional allure.

## Introduction

Invasive species threaten global biodiversity, compromise ecosystem integrity, and inflict non-negligible costs on various economic sectors, e.g., agriculture (Bradshaw et al., 2016; Paini et al., 2016). In Southeast Asia, invasive species cause losses to agri-food production, human health, and the environment worth US$25.8–39.8 billion per year (Nghiem et al., 2013). Similarly, invasive species cause more than US$ 100 billion in yearly losses in China (Paini et al., 2016), thus impacting the livelihoods of millions of smallholder farmers and reversing gains in rural development. Though some species exhibit a restricted distribution in China’s southeastern provinces (Xu et al., 2014), others have proliferated across the nation.

One notorious invasive species is the coconut black-headed caterpillar, *Opisina arenosella* Walker (Lepidoptera: Xylorictidae) (BHC). First described in India and Sri Lanka during the mid-19th century, *O. arenosella* has recently invaded several Asian countries, including Myanmar, Bangladesh, Thailand, Malaysia, Vietnam, and Pakistan (Perera et al., 1988; Muralimohan et al., 2007; Lyu et al., 2013). In Thailand, *O. arenosella* was first reported in Prajuabkirikhan province in 2007. It has since spread to 29 provinces nationwide, primarily affecting coconut production. In 2013, *O. arenosella* was equally recorded from Wanning City (Hainan), spreading to Guangdong, Guangxi, and Fujian in China’s mainland (Yan et al., 2013). In 2020, *O. arenosella* was recorded in Vietnam’s Ben Tre province and had since spread throughout the Mekong Delta (Le et al., 2022). Black-headed caterpillar larvae construct silk-covered feeding galleries within which they consume the leaf lamina of palms, thus resulting in a reduction of the photosynthetic area of affected plants. Extensive feeding damage can lead to the defoliation and death of entire palm trees (Lu and Wang, 2013). While BHC adults exhibit a strong flight ability and dispersal potential (Liu et al., 2014a), its larvae are exclusively found within palm fronds, on palm leaves, and young fruits. In several affected countries, large coconut plantations were destroyed following the arrival of *O. arenosella*. Though the BHC invasion initially triggered (unguided) the use of synthetic pesticides, a suite of non-chemical approaches have since been developed to sustain this species.

Here, it provide a comprehensive overview of *O. arenosella* taxonomy, biology, ecology, invasion history, and management across its native and invasive range. The work primarily focuses on non-chemical integrated pest management (IPM) methods and prevention strategies. These include crop diversification, pheromone-based monitoring and the use of (native, exotic) natural enemies for BHC biological control. Aside from reporting current achievements, we also define future prospects for a sustainable, ecologically-centered, and environmentally sound management of *O. arenosella* throughout Asia.

## Biology

### Host range

*Opisina arenosella* was earlier known as *Nephantis serinopa* Meyr. (described by Meyrick, 1908) and included under the family Cryptophasidae. Becher (1981) found that *Nephantis* and *Opisina* are monotypic genera as the type specimens deposited in the British Museum are conspecific. Hence, the name *Nephantis serinopa* was changed as *Opisina arenosella* and was placed within the subfamily Xyloryctinae under the Oecophoridae. More recent work however has unveiled how the Xyloryctidae family is an independent unit under the Gelechioidea (Nieukerken et al., 2011) which shares ancestry with the Oecophoridae. Hence, earlier studies place *O. arenosella* within the Oecophoridae (Cock and Perera, 1987; Mohan et al., 2010). Despite these historic changes in taxonomic status of the Xyloryctidae (i.e., either as subfamily or independent family), the genus *Opisina* has continually been placed under this taxonomic unit.

BHC is an oligophagous herbivore that is primarily associated with different palm species. Coconut palm (*Cocos nucifera* L.) is the primary host of BHC. Other host plants include wild date palm (*Phoenix sylvestris* L.), Chinese fan palm (*Livistona chinensis* (Jacq.) R. Br.), Washington palm (*Washingtonia robusta* H.Wendl.), betelnut palm or Areca palm (*Areca catechu* L.), royal palm (*Roystonea regia* (Kunth) O. F. Cook), sugar palm (*Arenga pinnata* (Wurmb) Merr.), butterfly palm (*Chrysalidocarpus lutescens* H.), foxtail palm (*Wodyetia bifurcata* K.), bottle palm (*Hyophore lagenicaulis* L), Alexandra palm (*Archontophoenix alexandrae* H.) and palmyra palm (*Borrassus flabellifer* L.) (Rao et al., 1948). Other host plants include date palm (*Phoenix dactylifera* L.) (Butani, 1975), talipot palm (*Corypha umbraculifera* L.) (Talati and Butani, 1988), sago palm (*Metroxylon sagu* Rottboell), Kithul palm (*Caryota urens* L.), doum palm (*Hyphaene thebaica* L.) (Lever, 1969), cabbage palm (*Oredoxa oleracea* Kurth), and Nipa palm (*Nypa fruticans* Wurmb.) in Vietnam (unpublished data). In addition to the above palm species, *O. arenosella* feeds upon banana (*Musa* spp.) (Manjunath, 1985) and sugarcane (*Saccharum officinarum* L.) (Muralimohan and Srinivasa, 2005). While BHC exhibits a clear oviposition and feeding preference on palmyra palm, royal palm, coconut palm and wild date palm (Cammell et al., 1990; Srinivasa et al., 1995), it also feeds upon banana in heavily infested coconut orchards i.e., when its primary host is depleted (Shameer et al., 2018). Under laboratory conditions, BHC equally completes its development on jack fruit and cashew nut (Kumara et al., 2015; Shameer et al., 2018) and its larvae consume leaves of maize, pineapple and rubber (Sukhirun et al., 2015). Overall, feeding damage is most pronounced on coconut, bismarck palm (*Bismarckia nobilis* Hildebrandt & H.Wendl.) and lipstick palm (*Cyrtostachys renda* Blume).

### Population dynamics

The coconut black-headed caterpillar passes through four consecutive development stages: egg, larva, pupa and adult. For Indian strains of *O. arenosella*, development times for the egg stage are 5-8 d, larval stage 42-48 d, pupal stage 12 d, while adult longevity ranges between 5-8 d at 26°C (Lyu et al., 2013; Muralimohan et al., 2013; Kumara et al., 2015).

Climatic variables affect BHC population dynamics in the field (Perera et al., 1988; Shameer et al., 2018), with low temperatures slowing or impeding population build-up (Lin et al., 2019). At low ambient temperature (e.g., January in India), the *O. arenosella* pre-oviposition period extends to 3-4 d (Muralimohan et al., 2013). During November, adult fecundity levels were highest with BHC adult females depositing an average of 231.1 ± 19.6 eggs (mean ± SE). Low temperatures during January lead to low egg eclosion rates (2.7%), resulting in the lowest number of first-instar larvae (4.17 ± 2.94 individuals) as compared to March (87.9 ± 14.9 individuals; Lin et al., 2019). The BHC attain the highest abundance levels during late summer i.e., July-November (Lin et al., 2019). In Mangalore (India), high summer temperatures favor the development of *O. arenosella* (Nadarajan and Channabasavanna, 1980), while high humidity and moderate temperatures are the main determinants of *O. arenosella* population build-up in Kerala (India) (Sathiamma et al., 1973). Conversely, in Karnataka (India), peak population levels are recorded during March-May and are affected by temperature, sunshine and rainfall (Way et al., 1989; Pushpalatha and Veeresh, 1995). In parts of its native range (e.g., Sri Lanka), the rare occurrence of *O. arenosella* outbreaks (i.e., affecting merely 0-1% of the local coconut crop) has been ascribed to the presence of various effective natural enemies (Way et al., 1989).

**Figure 1:**
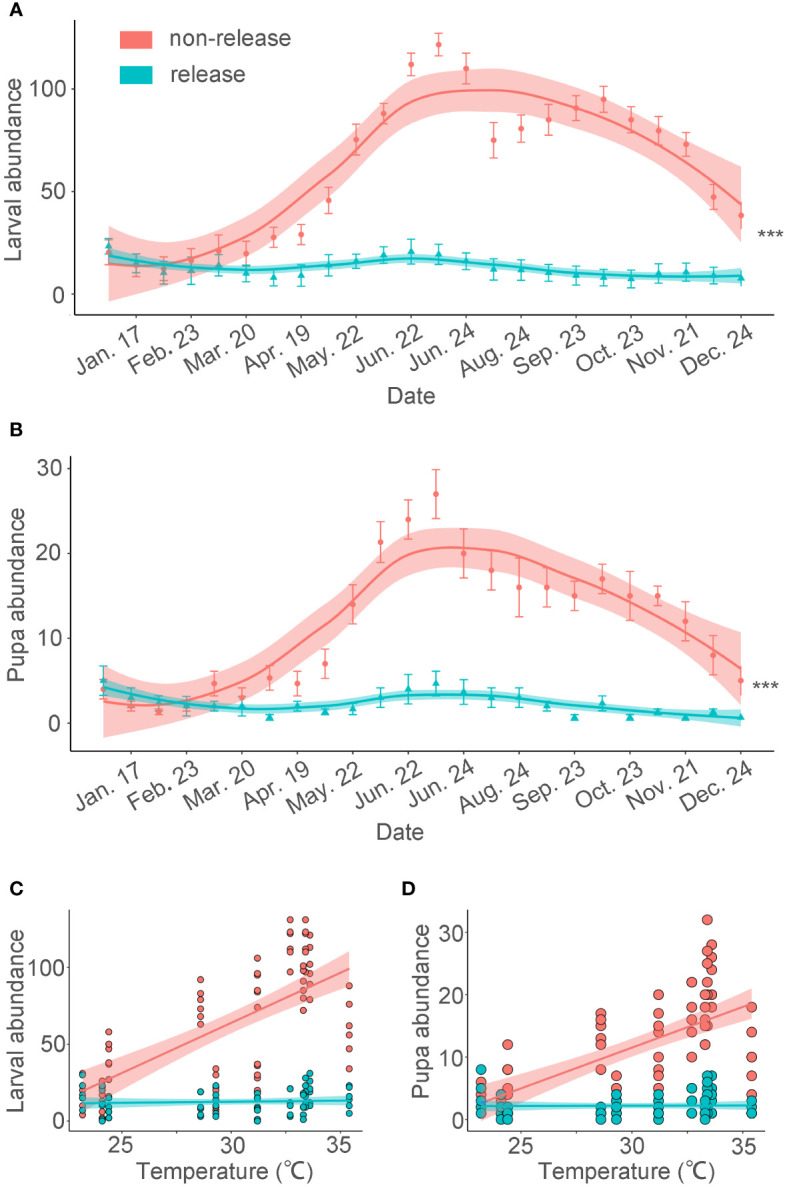
BHC larval and pupal abundance following augmentative releases of *Habrobracon hebetor* and *Chouioia cunea* in a coconut plantation. The following release rates were adopted: *C*. *cunea* were released at 10,000 individuals and *H*. *hebetor at* 5,000 individuals every two weeks. Releases were only conducted during January-June 2018 in Baodao village, Danzhou City, Hainan, China. Average BHC larval and pupa abundance (± SEM) per coconut tree is plotted on a monthly basis for the entire year (Wu, 2019). **(A, B)** (GLMM poisson,***p < 0.001). The abundance values are expressed per tree. The error bars are SE. The lines are loess curve line. **(C)** Linear regression captures the relationship between temperature and quantity (R^2^_non-release_=0.45, *p* <0.001; R^2^_release_=0.23, p = 0.55). **(D)** Linear regression explains the correlation between temperature and quantity (R^2^_non-release_=0.41, p <0.001; R^2^_release_=0.21, p = 0.89).

Upon its establishment in southern China, BHC exhibited similar seasonal dynamics as in its native range (**Figure 1**). Its overall abundance closely relates to temperature i.e., *O. arenosella* population size increases from April onwards and reaches outbreak levels in July (i.e., 121.9 ± 10.4 larvae per tree; Wu, 2019). On the other hand, infestation levels are low during January-May with minimum abundance in early February i.e., 13.6 ± 3.7 larva per tree. In Thailand, several overlapping *O. arenosella* generations co-occur in coconut fields and BHC attains peak abundance during December-April (i.e., local dry season). However, while there is ample information on *O. arenosella* during the above times of peak abundance and activity, its infestation levels (and host plant usage patterns) during times of lower abundance are rarely assessed (Shameer et al., 2018).

### Semiochemical communication

Gravid females respond to different volatile compounds or so-called semiochemicals. Following observations that BHC adults are attracted to odors from coconut leaves and larval frass (Kumara et al., 2015), linalool, acetophenone and limonene were identified as electro-physiologically active compounds. The composition of volatile organic compounds differs markedly between between infested and uninfested plants, and between different coconut varieties (Shameer et al., 2017). Also, field observations unveiled how *O. arenosella* males are attracted to young female moths (Chandrasiri and Fernando, 1997). Upon dissection of the pheromone glands of virgin *O. arenosella* females, gas chromatography coupled with electro-antennographic detection (GC–EAD) and mass spectrometry (GC–MS) identified (Z,Z,Z)-3,6,9-tricosatriene (Z3Z6Z9-23Hy) as its main physiologically active compound (Bhanu et al., 2018). GC-EAD assays showed strong male antennal activity following exposure to female pheromone gland extract and synthetic Z3Z6Z9-23Hy. In cage experiments, higher numbers of *O. arenosella* adults (93.2% being male) were caught in pheromone-baited traps (Bhanu et al., 2018). Exploratory work by Subaharan et al. (2005) reveals how olfactory conditioning possibly can enhance the efficacy of augmentation biological control (Kruidhof et al., 2019). These kairomones and sex pheromones constitute effective attractants for adult *O. arenosella* under field conditions, and potentially can be integrated into volatile-based monitoring or management approaches e.g., mating disruption or mass-trapping.

## Economic relevance and management

### Feeding damage and yield loss

In India, China or Thailand, *O. arenosella* regularly attains outbreak levels and has become a key pest of cultivated palm species (Borse, 2014). Though BHC affects oil palm in Thailand, it only reaches pest status on coconut *Cocos nucifera* L. Larvae of *O. arenosella* feed and defecate on the leaves and buds of various plant species, thereby interfering with photosynthesis. In cultivated palm species such as coconut, BHC feeding reduces the number of flower spikes, slows plant growth, and leads to premature fruit drop (Lever, 1969; Manjunath, 1985). Feeding damage is conspicuously different from that of the coconut hispine beetle *Brontispa longissima* (Gestro) (Coleoptera: Chrysomelidae); another limiting pest of regional importance (e.g., Wyckhuys et al., 2020). While *B. longissima* only affects young palm leaves, *O. arenosella* larvae feed on the old and new leaves alike, and equally attack buds and immature fruits (Lever, 1969; Manjunath, 1985). Following BHC feeding, palm leaves routinely wither and die. As such, BHC attacks can considerably reduce primary productivity of palm trees (Ramachandran et al., 1979), with a suite of negative socio-economic repercussions.

BHC can inflict considerable production losses in coconut (Rao, 1924; Jayaratnam, 1941; Nirula, 1956); a crop native to insular Southeast Asia and extensively cultivated across the region. Coconut is regularly called the “tree of life”, as its cultivation has important cultural and socio-economic facets, and sustains the livelihoods of countless smallholders across the Asia-Pacific. Coconut palm is the primary host of *O. arenosella*, and the larvae regularly consume more than 90% of the coconut leaves (Nirula, 1956). In Karnataka (India), *O. arenosella* affected 1.6 million coconut palm trees and caused extensive defoliation during the late 1990s (Shivashankar et al., 2000). In India’s Kerala State, crop losses up to 45% were reported one year after pest outbreaks; BHC feeding equally reduced bunch and leaf production by a respective 21 and 14% (Chandrika et al., 2010). After heavy pest infestation, yield levels are routinely restored by the fourth year (Mohan et al., 2010; Kumara et al., 2015). In newly invaded areas (e.g., Pahang, Malaysia), pest attack is often more severe than in its native range (Shameer et al., 2018). In those areas, BHC inflicts yield losses up to 60% in coconut palm plantations (Mahadi et al., 2019). Given betelnut palm’s economic and socio-cultural importance in several Asian countries (including China, India, Myanmar, Indonesia, or Papua New Guinea), BHC feeding damage on this crop can also have considerable impacts. Even though *O. arenosella* affects other cultivated crops such as banana (*Musa* sp.) (Manjunath, 1985), its impacts in terms of yield or production losses are considerably lower than on coconut and betelnut palm.

Following its invasion of Hainan (China) in 2013, *O. arenosella* has caused 5-40% losses in the local coconut sector (Lyu et al., 2013). These translate into US$ 18-147 million/year in direct economic costs, US$ 85-108 million/year in indirect costs, and US$ 1.1-7.9 million/year in prevention and control costs. Direct economic costs reflect BHC-induced yield declines or total crop loss, while indirect costs refers to off-site impacts capture cascading effects on service, transportation and processing industries (Yan et al., 2015). The above impacts for Hainan’s coconut sector are further compounded by US$160 million/year losses in the local betelnut industry. Pest-induced losses are thought to be more pronounced in unmanaged areas e.g., natural habitat, forested lands, roadsides, as compared to nurseries, commercial plantations, urban settings, or managed landscapes.

### Current management

Early detection is a first, critical step towards the effective containment, eradication and control of invasive pests such as BHC (e.g., Walter et al., 2021). Though routine monitoring in invasion-prone areas is essential to effectively manage this pest, visual detection is compromised by the cryptic feeding habits of *O. arenosella*. BHC caterpillars tend to feed in the canopy of palm trees often at heights beyond 10 m, where they inhabit silk-lined, frass-covered feeding galleries. The tallness of coconut palm makes it difficult to detect the infestation in the early stages.

Various (curative, preventative) management approaches are used to mitigate *O. arenosella* in its invaded and native range. In countries where BHC is endemic (i.e., Sri Lanka), its populations are regulated by resident natural enemies (Way et al., 1989). Under those conditions, *O. arenosella* outbreaks solely occur after ecological upsets as triggered by natural or human-mediated factors, e.g., insecticide use or hurricane damage. As such, there is rarely a need for curative management. In areas where BHC has been present for several decades such as India, both endemic and exotic natural enemies lower *O. arenosella* infestation levels. In India, Thailand and China, high rates of BHC biological control are attained through augmentative releases of various (egg, larval, pupal) parasitoids e.g., *Goniozus nephantidis* (Muesbeck), *Brachymeria nephantidis* Gahan, *Trichogramma embryophagum* (Hartig) (Raghwani et al., 1997). The above parasitoids attack the larval, pupal and egg stage, respectively. Given that parasitoid releases are routinely alternated with insecticide spray applications in Thailand, it is essential to closely scrutinize the necessity, efficacy and cost-effectiveness of such combined efforts (Chomphukhiao et al., 2018).

During initial stages of the BHC invasion e.g., in China, local coconut growers generally resorted to synthetic pesticides (Li et al., 2020). During the 1970s, Indian farmers applied highly hazardous organophosphates and organic phosphorus to control *O. arenosella* (Sathiamma and Kurian, 1972). In addition to foliar spray applications, monocrotophos was also injected into coconut trunks and exhibits prolonged systemic action against BHC larvae in the palm crown (Kanagaratnam and Pinto, 1985). Over the ensuing decades, organophosphates were gradually replaced by less hazardous compounds e.g., the chitin synthesis inhibitor (CSI) diflubenzuron (Sundaramurthy and Santhanakrishnan, 1979). Though abamectin and emamectin benzoate exert good insecticidal activity against BHC larvae in China, repeated spray applications only attain slight reductions in *O. arenosella* infestation levels i.e., from 12.8% to 3.3% in infestation hotspots (Liu et al., 2014b). In Thailand, emamectin benzoate is recommended for trunk injection (Punyawattoe et al., 2016), while foliar sprays of flubendiamide, chlorantraniliprole, spinosad, lufenuron are often used on young BHC-affected coconut palms. Trunk injections with the water-soluble biopesticide Soluneem equally provide effective control of *O. arenosella* larvae in India (Shivashankar et al., 2000). Yet, irrespective of their exact nature, biocidal compounds that are injected into the palm trunk likely affect non-target herbivores and beneficial biota that feed upon palm leaves, (extra-)floral nectar or pollen. Such organisms include insect or vertebrate pollinators and biological control agents such as predatory ants (Way et al., 1989; Chakravarthy and Thyagaraj, 2012). The above impacts are possibly exacerbated for broad-spectrum insecticides and neonicotinoids (Van der Sluijs et al., 2015).

Yet, over the past decade, multiple non-chemical tactics and preventative IPM measures have been locally devised, validated, and adopted. For example, Thai coconut growers increasingly use endotoxins of *Bacillus thuringiensis* Berliner (IPPC, 2017). Soil drenches with mixtures of garlic and azadirachtin effectively reduce BHC population levels in India (Chandrashekharaiah, 2013). Sanitary practices such as the removal and burning of BHC-affected leaves also lower infestation levels. Further, light-traps (i.e., water-filled trays placed below a halogen light) are widely used to mass-trap *O. arenosella* adults in India (Muralimohan et al., 2007). In the latter, BHC adults respond strongly to 365-368 nm wavelengths from 11:00 pm to 2:00 am (Yan et al., 2015). Lastly, the laboratory-based identification of BHC kairomones and pheromones has facilitated the development of volatile-based mass-trapping, mating disruption, and monitoring approaches. Following the identification and synthesis of *O. arenosella* pheromones (Bhanu et al., 2018), pheromone traps have been widely used to monitor BHC population levels and guide management interventions in coconut plantations (Bhanu et al., 2018). The deployment of pheromone traps on its own can reduce BHC population levels by 50-94% (Chandrashekharaiah, 2013). Hence, a myriad of sustainable mitigation strategies are available for BHC-afflicted coconut growers across the region.

## Assessing the invasion of Southeast Asia

The biological invasion process can be organized into consecutive phases, i.e., arrival, establishment, and spread of the invasive species (Mack et al., 2002). Not only does this step-wise process help to quantitatively assess the likelihood, magnitude, and ensuing impact of a given invasion, but it also provides a suitable framework to chronicle the Asia-wide invasion of *O. arenosella* over the past decades.

## Invasion history

**Figure 2:**
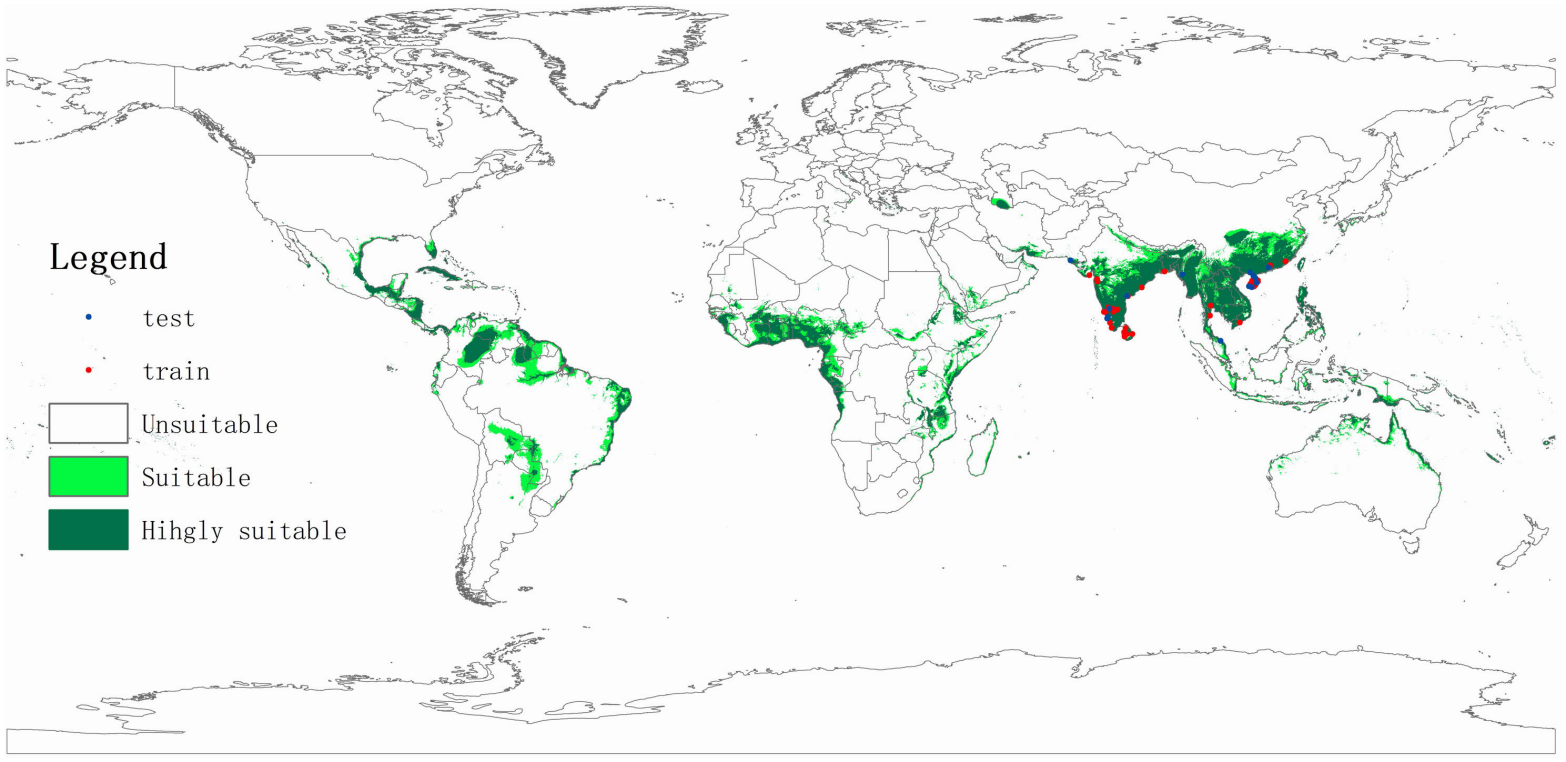
Potential geographic distribution of *Opisina arenosella* as predicted through climate-based niche modeling. Suitable areas are delineated using Maxent (Phillips and Dudik, 2008), using occurrence records drawn from Lyu et al. (2018), Wu (2019), and the Centre for Agriculture and Bioscience International (CABI; www.cabi.org). Nineteen bioclimatic variables, averaged over 1950-2000 and with a spatial resolution of 2.5 arc min (*c*. 5 km), were downloaded from the WorldClim database (http://www.worldclim.org). A digital elevation model at 1 km^2^ (∼30 arc s) resolution was obtained from the National Geophysical Data Center (www.ngdc.noaa.gov).

The coconut black-headed caterpillar likely originates in Sri Lanka and (possibly) parts of southern India (Nirula, 1956; Cock and Perera, 1987; Singh and Rethinam, 2006; Kumara et al., 2015). In 1909, *O. arenosella* was first recorded in coastal areas of southern India – where it was first reported as an (economically relevant) pest in 1920 (Rao et al., 1948). Since the late 1900s and early 2000s, BHC successively invaded countries such as Myanmar (Ghosh and Abdurahiman, 1985), Bangladesh (Alam, 1962), Thailand, and Malaysia (Lyu et al., 2013). While historical records (i.e., Davis and Sudasrip, 1982) signal the presence of *O. arenosella* in Indonesia, these accounts are likely to be erroneous (Cock and Perera, 1987). Nevertheless, its invasion is likely to occur in northern parts of Indonesia (e.g., Aceh, North Sumatra, Riau) through BHC-affected parts of Malaysia or Thailand. BHC was first recorded in Thailand in 2007, where it presently exhibits a restricted geographical distribution and finds itself under control (IPPC, 2017). In August 2013, *O. arenosella* was also discovered in Wanning City (Hainan, China), from where it likely spread to Guangxi and Guangdong provinces (Lu and Wang, 2013; Li et al., 2014). At present, *O. arenosella* is widely distributed in Hainan island though exhibits variable levels of abundance and crop damage (Yan et al., 2015; **Figure 2**). In China, *O. arenosella* presently occurs south of 23.5°N (Lyu et al., 2013); in India, the northernmost record of *O. arenosella* is in Delhi at a latitude of 28.6°N (Jalali et al., 2005). Lastly, in recent years, BHC has also been officially reported from southern Vietnam (Le et al., 2022).

Several aspects of the species’ biology and ecology facilitate its inter- and intra-country spread. In addition to BHC adults’ strong (nocturnal) flight capacity, the species benefits from the human-mediated movement of its (palm) host plants and coconut products. BHC larvae and pupae are occasionally found on harvested coconuts in affected areas. Harvested (fresh) coconuts and traded ornamental plants thus potentially act as a vehicle for the initial invasion inoculum. For example, China annually imports 2 billion coconuts from ASEAN countries and regularly intercepts invasive pests in trade consignments from countries such as Thailand or Vietnam (He et al., 2019). The oligophagous feeding habits and widespread distribution (or extensive cultivation) of various *O. arenosella* host plants further aid its establishment and spread. Aside from the coconut palms that line the coastlines of all tropical Asian countries, *Phoenix sylvestris* Roxb, *Livistona chinensis* (Jacq.) R. Br., *Washingtonia robusta* H. Wendl. And betelnut palms are omnipresent in its countryside. Lastly, climatic similarities between South and Southeast Asian countries and the BHC region of origin (i.e., Sri Lanka) greatly favor the species’ establishment, spread, and economic impact. Climate niche modeling shows how several key palm-growing areas in Asia, Africa and Latin America have high degrees of climate similarity with the current BHC-affected areas (**Figure 2**). These include all African countries lining the Gulf of Guinea, Brazil’s Roraima and Para states, the Colombian and Venezuelan Llanos, and the coastline of most Central American countries. To prevent BHC establishment and spread in these areas, a suite of quarantine measures can be implemented, e.g., careful inspection of traded plant products and the deployment of pheromone traps in or near ports of entry. Climate change possibly will expand BHC geographical distribution, alter its seasonal dynamics and raise its infestation levels, while extreme weather events e.g., drought or flooding may weaken host tree defenses. National and international entities (e.g., FAO-IPPC) can help to devise, implement and enforce preventative regulations regarding the inter- and intra-country movement of live palms. Given the tangible risk of intercontinental spread, a globally-coordinated strategy and close (coconut) industry engagement are imperative to swiftly and effectively deploy best-bet solutions e.g., as evidenced in the fiber and pulpwood sector (Wingfield et al., 2015; Hurley et al., 2016).

## Ecology

Throughout its native and invasive range, a speciose community of (vertebrate, invertebrate, microbial) natural enemies is associated with different BHC development stages (Cock and Perera, 1987; Lyu et al., 2018). For example, more than 40 species of parasitoids and over 50 predator species attack *O. arenosella* in India (Pillai and Nair, 1993; Winotai, 2014). These beneficial organisms contribute to *O. arenosella* population regulation, and several are used for biological control. While the resident natural enemy community has been well-documented in India and Sri Lanka, relatively few descriptive (and basic or applied ecological) studies have been conducted in other BHC-invaded countries such as Myanmar, Thailand, Malaysia or China.

## Insect parasitoids

A diverse parasitoid complex is associated with BHC, comprising members of the Braconidae, Eulophidae, Chalcidae, Bethylidae, Ichneumonidae, Tachinidae, and Trichogrammatidae (Cock and Perera, 1987; **Table 1**). These involve egg parasitoids such as *Trichogrammatoidea armigera* Nagaraja, *Trichogramma evanescens* Westwood or *Trichogramma pretiosum* Riley (Jalali et al., 2002); larval parasitoids such as *Goniozus nephantidis* Muesebeck, *Apanteles taragamae* Viereck or *Habrobracon hebetor* (Say) (Ghosh and Abdurahiman, 1988; Archna et al., 2013); and pupal parasitoids such as *Tetrastichus howardi* Olliff, *Brachymeria nosatoi* Habu or *Elasmus nephantidis* Rohwer (Kurian and Sathiamma, 1970). Multi-parasitism is frequently recorded for *Trichospilus diatraeae* Cherian & Margabandhu, *Tetrastichus israeli* Mani & Kurian, *Trichospilus pupivorus* Ferriere, and *Meteoridea hutsoni* Nixon (Pillai and Nair, 1993). Among the above BHC parasitoids, *Trichogramma chilonis* Ishii, *Trichogramma embryophagum* Hartig, *A. taragamae*, *G. nephantidis*, *B. nosatoi*, and *E. nephantidis* regularly attain high levels of parasitism and contribute substantially to *O. arenosella* biological control (Jalali et al., 2005; Venkatesan et al., 2007; Wu et al., 2018a). Particularly in its invasive range e.g., China, generalists constitute the bulk of the parasitoid complex. For example, *H. hebetor* and the eulophid *Chouioia cunea* Yang are important BHC parasitoids but equally affect several other (lepidopteran) species in agricultural and natural habitats including the invasive fall webworm, *Hyphantria cunea* Drury (Lepidoptera: Erebidae) (Xin et al., 2017; Wu et al., 2018b).

**Table 1:** Parasitoids associated with different developmental stages of *Opisina arenosella* in Asia (*E* egg, *L* larvae, *P* pupae, *A* adult).

Order	Family	Species	E	L	P	A	Location
Hymenoptera	Trichogrammatidae	*Trichogramma pretiosum*	x				I
		*Trichogramma brasiliense*	x				I, S
		*Trichogramma chilonis*	x				I
		*Trichogramma evanescens*	x				I
		*Trichogramma embryophagum*	x				I, T
		*Trichogramma dendrolimi*	x				I
		*Trichogramma japonicum*	x				I
		*Trichogramma achaeae*	x				I
		*Trichogrammatoidea armigera*	x				I
		*Trichogramma bactrae*	x				I
		*Trichogramma* sp.	x				I
		*Trichogramma minutum*	x				S
	Braconidae	*Apanteles* sp.					T
		*Apanteles taragamae*		x			I
		*Meteoridea hutsoni*		x			I
		*Bracon hebetor*		x			I, C, T
		*Bracon brevicornis*		x			I, S, T
		*Bracon adoxophyesi*		x			C
		*Habrobracon hebetor*		x			T
	Ichneumonidae	*Eriborus trochanteratus*		x			I, S
		*Goryphus* sp.			x		I
		*Xantopimpla* sp.			x		I
		*Xanthopimpla nana nana*			x		I
		*Xanthopimpla punctata*			x		I
	Tachinidae	*Bessa remota*		x			M, T, S
		*Stomatomyia bezziana*		x			I
	Bethylidae	*Goniozus nephantidis*			x		I, C, T
	Eulophidae	*Elasmus nephantidis*		x			I
		*Tetrastichus israeli*			x		I
		*Tetrastichus howardi*			x		C
		*Trichospilus pupivorus*			x		M, T
	Chalcididae	*Brachymeria* sp.			x		T
		*Brachymeria euploeae*		x	x		I,T
		*Brachymeria nosatoi*			x		I, C
		*Brachymeria euploeae*			x		I
		*Brachymeria nephantidis*			x		I, T
		*Brachymeria lasus*			x		I
		*Brachymeria excarinata*			x		I
		*Elasmus nephantidis*			x		I, S
		*Antrocephalus hakonensis*			x		I
		*Antrocephalus mitys*			x		S
		*Chouioia cunea*			x		C

Occurrence records are drawn from Jalali et al. (2002), Lyu et al. (2013), and Winotai (2014). Countries with a given parasitoid from O. arenosella are indicated (I Indian, S Sri Lanka, C China, M Malaysia, T Thailand). This listing is not intended to be exhaustive and is based on available literature sources.

The primary BHC egg parasitoids are *Trichogramma* spp. Under laboratory conditions, *Trichogramma bactrae* Nagaraja, *T. brasiliensis* Ashmead, *T. chilonis* Ishii, *T. embryophagum* Hartig, *T. dendrolimi* Matsumura, *T. japonicum* Ashmead, and *T. achaeae* Nagaraja and Nagarkatti attain the highest parasitism levels (i.e., 82-98%; Jalali et al., 2005). Several of these parasitoids (e.g., *T. embryophagum*, *T. japonicum*) are used for augmentative biological control of *O. arenosella* in India, China or Thailand (Jin et al., 2019). This practice involves the inoculative or inundative release of laboratory-reared parasitoids in BHC-affected orchards to either supplement resident populations in early season (i.e., well ahead of an outbreak) or directly achieve pest suppression.

Among the suite of larval parasitoids, the gregarious ectoparasitoid *G. nephantidis* is the dominant species in India (Dharmaraju, 1962; Cock and Perera, 1987). This parasitoid attacks late larval development stages i.e., instars 5-8 (Shameer et al., 2018). In western India, *G. nephantidis* reaches 30% parasitism under field conditions (Kapadia and Mittal, 1993). Meanwhile, *A. taragamae* mainly parasitizes young BHC larvae and reaches high parasitism levels in India’s coastal areas during the summer and inland during winter (Sujatha and Singh, 2004; Mohan and Sathiamma, 2007). In Hainan (China), *H. hebetor* and *Bracon adoxophyesi* Mimanikawa parasitize BHC larvae (Huang et al., 2017). The latter species acts as an ectoparasitoid on older larvae and pre-pupae of *O. arenosella*, while its ovipositor-probing kills young BHC (Lin et al., 2018). In Thailand and India, *Habrobracon hebetor* (Say) plays an essential role in BHC biological control (Nasser and Abdurahiman, 2001; IPPC, 2017; Chomphukhiao et al., 2018). To complement the action of endemic parasitoids, certain species (e.g., the tachinid *Bessa remota* Aldrich) were introduced into India (Jayanth and Nagarkatti, 1984). Overall, the BHC larval parasitoid complex’s exact composition and aggregate parasitism level is shaped by agro-ecological and climatic variables. While *G. nephantidis*, *A. taragamae* and *H. hebetor* are key larval parasitoids of *O. arenosella* in India’s Bangalore (Nadarajan and Channabasavanna, 1980), this is not the case in Kerala State (Pillai and Nair, 1993; Pushpalatha and Veeresh, 1995). Given the above, assessing the environmental determinants of species abundance and parasitism efficacy is crucial.

Regarding pupal parasitoids, *T. howardi*, *B. nosatoi*, *E. nephantidis* (pre-pupal) and *C. cunea* are the dominant species. Several of the above species (e.g., *T. howardi*) are generalists that neither prefer BHC pupae nor attain adequate development on this species (Baitha et al., 2003). Conversely, the generalist *C. cunea* reaches high parasitism levels on *O. arenosella* in China (Yang, 1989; Zheng et al., 2012; Lyu et al., 2018). In Gujarat (India), *Brachymeria nephantidis* Gahan and *Brachymeria lasus* (Walker) are recorded, with the former species assuming a pivotal role in BHC biological control (**Table 1**). In Hainan (China), *Brachymeria nosatoi* Habu was recently recovered from BHC pupae (Lyu et al., 2018; Wu et al., 2020), while laboratory assays have been conducted to evaluate the efficacy of the eulophid *Trichospilus pupivorus* in Malaysia (Mahadi et al., 2019).

The life history and (nutritional) ecology of several of the above parasitoids have been studied, with the primary aim of developing laboratory mass-rearing systems. Considering how several BHC parasitoids are generalist species (e.g., *B. nosatoi*), they can be easily maintained on larvae or pupae of stored product pests such as the rice moth *Corcyra cephalonica* (Stainton) or the Angoumois grain moth *Sitotroga cerealella* (Olivier) (Shameer and Mohan, 2002). When *G. nephantidis* is raised on the wax moth *Galleria mellonella* L., its fitness (i.e., development period, fecundity, parasitism rate, adult weight) is not negatively affected. When reared on *G. mellonella* or *C. cephalonica*, biological parameters of *G. nephantidis* remained unaffected (Mohan and Shameer, 2003). Meanwhile, when using *C. cephalonica* larvae as an alternative host, *G. nephantidis* can be raised in larger quantities (Venkatesan et al., 2009). Larval parasitoids such as *H. hebetor* can also be maintained on *C. cephalonica* and *S. cerealella* (Mohan and Sathiamma, 2007). Adult nutrition is another key parameter in mass-rearing, and the impact of various carbohydrate sources on *T. pupivorus* survival, longevity, and reproduction has been assessed (Mahadi et al., 2019). For *G. nephantidis*, experimental work in Malaysia has shown how 30% honey-water solution is a suitable food source (Hariprasad and Venkatesan, 2006). Similarly, by characterizing *H. hebetor* life history (Lyu et al., 2018) and defining optimum nutritional and environmental conditions for its development, mass-rearing schemes were developed for this species.

Several species of hyperparasitoids are recorded from the *O. arenosella* invasive and native range (Ghosh and Abdurahiman, 1985). Any given primary parasitoid is associated with 1-10 hyperparasitoid species including *Pediobius imbreus* Walker, *Eurytoma braconidis* Ferriere, *Aphanogmus manilae* Ashmead or *Aphanogmus goniozi* Dessart (Ghosh and Abdurahiman, 1985). Some of these species (e.g., *Pediobius imbreus*; Sujatha and Singh, 2003) exhibit high levels of parasitism of the primary parasitoids (Ghosh et al., 1993) and lower parasitism of *Bracon brevicornis* Wesmael by 14% during summer (Sujatha and Singh, 2003). While hyperparasitoids can stabilize ecosystems, they can also interfere with biological control (Boivin and Brodeur, 2006; Tougeron and Tena, 2019). Hence, it is essential to gain a profound understanding of the make-up of the hyperparasitoid community, assess its environmental determinants and gauge its net contribution to BHC pest management.

## Arthropod predators

In BHC-affected areas, a multitude of (vertebrate, invertebrate) predators has been observed, including birds, spiders, pirate bugs, assassin bugs, and ground beetles (Cock and Perera, 1987; **Table 2**). In Sri Lanka, at least 15 ant species inhabit palm crowns, and several of these (i.e., *Monomorium floricola* (Jerdon), *Paratrechina longicornis* Latreille, *Crematogaster* spp.) prey upon *O. arenosella* egg masses (Way et al., 1989). Other important BHC predators include the anthocorid *Cardiastethus exiguus* Poppius, the lacewing *Mallada astur* Banks, the coccinellid *Jauravia* sp., the rufous treepie *Dendrocitta vagabunda parvula* (Latham) and the ground beetle *Parena nigrolineata* Chaudoir (Narayanan and Veenakumari, 2003; Lyla et al., 2006). Among these predators, various ant species and *C. exiguus* seemingly play a central role in *O. arenosella* biological control.

**Table 2:** Arthropod predators associated with different developmental stages of *Opisina arenosella* in Asia (*E* egg, *L* larvae, *P* pupae, *A* adult).

Order	Family	Species	E	L	P	A	Location
Diptera	Asilidae	*Astochia* sp.				x	S
Neuroptera	Chrysopidae	*Ankylopteryx octopunctata*		x	x		I
		*Brinckochrysa scelestes*		x			I
		*Mallada astur*		x			I
Hemiptera	Anthocoridae	*Cardiastethus exiguus*	x	x			I, T
		*Orius* sp.	x	x			I
	Reduviidae	*Sphedanolestes aurescens*	x	x			I
	Pentatomidae	*Eocanthecona furcellata*					
Coleoptera	Carabidae	*Calleida splendidula*		x	x		I
		*Creagris labrosa*		x	x		I
		*Parena nigrolineata*		x	x		I
	Coccinellidae	*Jauravia pubescens*	x	x			I
		*Menochilus sexmaculata*	x	x			I
		*Micraspis discolor*					I
		*Propylea fallax*	x	x			I
	Prionoceridae	*Idgia dimelaena*					I
Dermaptera	Chelisochidae	*Chelisoches morio*	x	x	x	x	T
Hymenoptera	Formicidae	*Crematogaster* sp.	x				S
		*Monomorium floricola*	x				S
		*Monomorium* sp.	x				S
		*Oecophylla smaragdina*	x				S
		*Paratrechina longicornis*	x				S
		*Technomyrmex albipes*	x				S
Passeriformes	Corvidae	*Corvus splendens*		x			S
		*Dendrocitta vagabund*a		x			I
Araneae	Salticidae	*Marpissa tigrine*		x	x		I
		*Hyllus* sp.		x			S
		*Plexippus* sp.		x			S
		*Rhene indicus.*		x			I
		*Rhene* sp.		x	x		S

Records are drawn from multiple literature sources. Countries with a given parasitoid from O. arenosella are indicated (I Indian, S Sri Lanka, T Thailand). This listing is not intended to be exhaustive and is based on available literature sources.

For several species, predation and development rates have been assessed under laboratory conditions, and some initial field-level assays have been carried out (Nasser and Abdurahiman, 2001). In tropical tree-based ecosystems, ants provide a suite of ecosystem services, including natural biological control (Drummond and Choate, 2011; Griffiths et al., 2018). Through a set of elegant manipulative assays, Way et al. (1989) showed how different ant species remove (sentinel) egg masses at rates of 50% (6 h) up to 95% (24 h). Exclusion assays revealed how (diurnal) ant-mediated predation accounts for 75-98% BHC egg mortality. This compares to spiders, which provide less than 5% egg predation. Small-sized ant species are particularly important, as they forage inside BHC larval feeding galleries. Way et al. (1989) call for further investigation to assess the determinants of egg predation by certain ant species (e.g., *M. floricola*), while accounting for the effects of climate, nutritional resources, and inter-species competition. Other species such as *C. exiguous*, *M. astur*, *Jauravia* sp., spiders, and ants equally prey upon early-instar larvae of *O. arenosella* (Mohan et al., 2010). However, intraguild predation e.g. between *G. nephantidis* and *C. exiguus* may influence pest population dynamics and the overall efficacy of natural or augmentation biological control (Velasco-Hernandez et al., 2021). In southern Vietnam, (unidentified) earwigs are commonly associated with *O. arenosella*, and their relative contribution to BHC biological control is currently being assessed (unpublished data). Lacewings such as *M. astur* prey upon BHC eggs and larvae in larval feeding galleries within the canopy of 30 m high trees (Sujatha and Singh, 2003). Under laboratory conditions, *M. astur* nymphs consume large numbers of newly hatched BHC larvae (Sujatha and Singh, 2003). Though the ground beetle *P. nigrolineata* preys upon *O. arenosella*, its contribution to BHC biological control is considered marginal (Pushpalatha and Veeresh, 1995). Yet, as Cock and Perera (1987) recommended, research is urgently needed to determine field-level incidence, activity patterns, or BHC predation rates of the resident (generalist, specialist) predators in different agro-ecological and geographical contexts.

## Entomopathogens

Several microbial agents have been isolated from BHC individuals in the field, primarily evaluated in India. For instance, the pathogenicity of multiple nuclear polyhedrosis viruses (NPVs) has been assessed under laboratory conditions (Narayanan and Veenakumari, 2003). One specific NPV strain causes widespread larval mortality in Kerala (India), with death occurring 3-8 days after infection (Moore, 2001). Similarly, a highly infectious strain of *Bacillus thuringiensis* (Bt) has been isolated from dead *O. arenosella* larvae in Hainan, China (Sun et al., 2016). In India, two field-collected isolates of *Metarhizium anisopliae* are pathogenic to *O. arenosella* larvae (Rachappa, 2003), and the efficacy of *Paecilomyces farinosus* (Dickson ex Fries) strains has been assessed in the laboratory (Kuruvilla and Jacob, 1980). So far, no Protozoa or entomo-pathogenic nematodes have been reported from *O. arenosella*. Overall, considerably more work is needed to discover, describe and deploy entomo-pathogenic fungi, bacteria and viruses for BHC management.

## Biological control

Biological control involves the effective harnessing of biodiversity for pest management. Vertebrate, invertebrate, and microbial biota can all be employed under three different types of strategies i.e., conservation, augmentation, and classical (or importation) biological control (Bale et al., 2008; Heimpel and Mills, 2017). Conservation biological control (CBC) entails the deliberate (field, farm- or landscape-level) conservation of naturally occurring beneficial organisms for the management of native or invasive pests (Barbosa, 1998; Wyckhuys et al., 2013; Gurr et al., 2017). This can be achieved by manipulating the broader agro-ecosystem, eliminating (anthropogenic) disturbances such as broad-spectrum insecticide use, or providing shelter, non-prey food (e.g., floral nectar, pollen), and alternative prey or host items (Landis et al., 2000; Gurr et al., 2017). CBC strategies can also be geared towards bolstering indirect ecological interactions, e.g., apparent competition through shared generalist parasitoids (Shameer et al., 2018). Classical or importation biological control (IBC) entails the judicious selection, importation, and release of (host-specific) exotic natural enemies for the control of invasive pests (Bach, 1962; Heimpel and Cock, 2018; Wyckhuys et al., 2020). Over time, IBC has become a safe, efficacious, and environmentally-sound pest management strategy that has enabled the long-term suppression of multiple high-profile pests in the Asia-Pacific (Bach 1962; Wyckhuys et al., 2020). Lastly, augmentation biological control (ABC) entails the periodic release of (mass-produced) natural enemies in an inoculative or inundative fashion (Bale et al., 2008). This form of biological control has received considerable attention and has proven to be a desirable, cost-effective management approach in Sri Lanka, India, Thailand, and China. Each of these forms of biological control is presently used in different parts of the (invasive, native) range of *O. arenosella* and involves the (scientifically guided) manipulation of tens of biological control agents or so-called natural enemies.

## Conservation biological control

Observational studies and food web analyses in Kerala (India) have shown how *O. arenosella* is attacked by 23 parasitoid species residing within local agro-ecosystems. By establishing intercrops (e.g., cucumber, mulberry, sugarcane, key lime, papaya) that provide alternative hosts for those shared parasitoids, one potentially can raise parasitoid abundance throughout the year and lower *O. arenosella* infestation pressure (Shameer et al., 2018).

Aside from characterizing the plant- or habitat associations of specific BHC parasitic wasps, further insights need to be gained into their nutritional ecology. Similarly as for other crops x pest systems, laboratory and field trials can unveil the relative role of flower visitation and nectar foraging by parasitic wasps such as *G. nephantidis* (Fiedler and Landis, 2007; Zhu et al., 2013). These assays should involve the multiple flowering epiphytes that inhabit the canopy of tropical palm species (Zotz and Vollrath, 2003) and understory plant species. Equally, the fitness implications of homopteran honeydew consumption for resident natural enemies remains to be empirically assessed (Wackers et al., 2008; Wyckhuys et al., 2008). By thus characterizing feeding patterns of key natural enemies, CBC schemes can be defined that involve establishing or conserving flowering plant species or palm-inhabiting homopterans in (natural, man-made) ecosystems across Asia. Ths type of nutritional ecology research possibly can center upon one or more generalist parasitoids.

CBC programs can equally target the predaceous ants or the anthocorid *C. exiguus* that inhabit the canopy of palm species. As for *C. exiguus*, one needs to assess its diet, predation behavior, and fitness implications of feeding upon certain prey and non-prey items, e.g., pollen – a valued alternative food source for many anthocorids (Cocuzza et al., 1997). Considering how ants account for 75-98% of egg predation, it has been hypothesized that BHC outbreaks -especially in its native range- are triggered by synthetic insecticide applications (Wood, 1971). In the highly diverse and comparatively stable tree cropping systems of tropical Asia, one potentially can bolster BHC biological control by refraining from foliar spray applications or trunk injections with systemic products. As above, ant-mediated biological control can also be raised through the provision of natural or artificial sugar sources (Perez-Rodriguez et al., 2021) or by adapting crop management schemes (Drummond and Choate, 2011).

## Classical biological control

Over the past half-century, at least 11 species of parasitoids have been introduced for *O. arenosella* management in other Asian countries (**Table 3**) though most have failed to establish. Some failures can be ascribed to the loss of parasitoid genetic diversity or searching ability during prolonged periods of laboratory testing, e.g., as observed with *Eriborus trochanteratus* (Morley) (Hymenoptera: Ichneumonidae) maintained on *C. cephalonica* as factitious host (Cock and Perera, 1987). Experiences with *T. israeli* and *Trichogramma minutum* Riley further underline the necessity to source species from the correct host and habitat. During 2012-2018, *G. nephantidis* and *B. nephantidis* were released in Thailand, where augmentative releases of the former species now provide high levels of BHC biological control (IPPC, 2017). A formal *ex-post* impact assessment of the *G. nephantidis* introduction into Thailand is worthwhile, as such, can characterize its (socio-economic) contribution to the country’s coconut palm sector.

**Table 3:** Geographical location and fate of historic classical biological control efforts against the coconut black-headed caterpillar, *Opisina arenosella* in Asia.

Order	Family	Species	Origin	Release area	Fate
Diptera	Tachinidae	*Bessa remota* (Aldrich)*	Fiji, Malaysia, Myanmar	India, Sri Lanka	F
		*Stomatomyia bezziana* Baranoff	Sri Lanka	India	E,L
Hymenoptera	Bethylidae	*Goniozus nephantidis* (Muesebeck)	Sri Lanka or India	Thailand	E
	Braconidae	*Bracon brevicornis* Wesmael	India	Sri Lanka	E,L
	Chalcididae	*Anthrocephalus pandens* Walker	India	Sri Lanka	F
		*Brachymeria nephantidis* Gahan	Sri Lanka	Thailand	F
	Eulophidae	*Tetrastichus israeli* (Mani & Kurian)*	India	Sri Lanka	F
		*Elasmus nephantidis* Rohwer	India	Sri Lanka	F
	Ichneumonidae	*Eriborus trochanteratus* (Morley)	Unknown	India	E
	Trichogrammatidae	*Trichogramma brasiliensis* (Ashmead)*	USA, India	Sri Lanka	F
		*Trichogramma minutum* Riley	Unknown, India	India, Sri Lanka	F

F, failure to establish on target host; E, established; L, marginal to low parasitism.

*Outspoken generalist.

Looking ahead, in-depth surveys in Bangladesh and Myanmar (i.e., as suggested by Cock and Perera, 1987) can help to identify new BHC-associated natural enemies. These could be included in future efficacy trials, host specificity assessments, and subsequent release programs. Additional work is required to discover and describe egg parasitoids in the native range of *O. arenosella* or to conduct a comprehensive (pre-release) screening of a Sri Lankan *E. trochanteratus* strain. Lastly, irrespective of the high parasitism rates of generalists such as *Stomatomyia bezziana* (Baranov) and *A. fumipennis* on BHC or related lepidopteran species (Cock and Perera, 1987), their involvement in future IBC carries substantial ecological risk and should not be further pursued. Instead, future endeavors should adopt globally established IBC guidelines and scientifically underpinned selection procedures, while consciously balancing the ecological risks and benefits of classical biological control (Barratt et al., 2010; Heimpel and Cock, 2018).

## Augmentation biological control

At least eight species of BHC parasitoids and predators are presently used in ABC schemes across tropical Asia. In Odissa (India), bi-weekly releases of laboratory-grown *H. hebetor*, *B. brevicornis*, and *G. nephantidis* provide 53-95% control of *O. arenosella* (Mohanty et al., 2000). In other parts of India, augmentative releases of *G. nephantidis* result in 90-100% BHC parasitism rates (Venkatesan et al., 2007), while combined releases of *H. hebetor* and *G. nephantidis* reduce BHC larval and pupal abundance by a respective 34-76% and 33-94% (Rao et al., 2018). In Bangalore (India), 76-89% reduction in *O. arenosella* infestation pressure is attained through periodic releases of *T. chilonis* and *T. embryophagum* (Jalali et al., 2005). In Kerala (India), repeated releases are made of mass-cultured *E. nephantidis* and *B. nosatoi* (Sathiamma and Kurian, 1972). Indian successes are mirrored by recent work in China, where laboratory-reared *C. cunea* and *H. hebetor* provide effective control of BHC and other invasive pests such as *Hyphantria cunea* (Drury) (Li, 1992; Xin et al., 2017). Following biweekly releases of *C. cunea* (8-10,000 individuals per district) and *H. hebetor* (4-5,000 individuals), infestation levels of *O. arenosella* larvae and pupae are reduced by a respective 79% (max. 91%) and 80% (max. 92%) (**Figure 1**). Similar successes have been achieved in Thailand with *G. nephantidis*, *B. brevicornis* and *B. nephantidis* (Winotai, 2014). In addition to these parasitoid releases, the anthocorid *C. exiguus* has also been used effectively with scheduled releases of 50-100 individuals per tree reducing the target pest population (Lyla et al., 2006). Lastly, the earwig *Chelisoches morio* (Fabricius) is reared on cat pet food and released at a rate of 8-32 individuals per tree, resulting in effective biological control of BHC (Suwongsaksri et al., 2020).

Dedicated research on mass-rearing methodologies, natural enemy physiology and ecology, and (in-field) release protocols underpinned the above successes. For example, field trials in Odissa (India) allowed defining the exact release rates for multiple species of parasitoids based upon BHC infestation levels (Mohanty et al., 2000). For single-species releases of *G. nephantidis*, rates of 10 wasps per tree yield acceptable levels of biological control (Venkatesan et al., 2007). Similarly, optimum release rates for egg parasitoids were defined as 1,000 individuals per tree (Jalali et al., 2005). Research on insect mass-rearing technology resulted in the development of an artificial diet for *O. arenosella* or enabled *C. exiguus* to be cultured on eggs of the alternative host *C. cephanolica* (Ballal et al., 2003). Similarly, research in India and Sri Lanka helped to improve the mass-rearing of the parasitoids *E. trochanteratus* (Perera, 1977), *Xantopimpla* sp. (Pillai and Nair, 1987), or *B. brevicornis* (Cock and Perera, 1987).

## Diversifying the IPM toolbox

Across Asia, *O. arenosella* is currently managed through sanitary practices, augmentation biological control, and chemical or biological pesticides delivered through aerial applications or trunk injection (Winotai, 2014). Even though crop sanitation is adequate, the selective removal (i.e., pruning) and burning of BHC-affected palm leaves are labor-intensive and thus comparatively expensive. Also, given the high levels of biological control provided by laboratory-reared and resident natural enemies (e.g., ants, parasitic wasps), there’s ample scope to drastically reduce synthetic pesticide use. Thus, tapping the potential of integrated pest management (IPM) or even agroecological pest management (APM) (Deguine et al., 2021; Wyckhuys et al., 2021; Deguine et al., 2023), one can prevent BHC population build-up, anticipate eventual pest outbreaks, and safeguard the resilience of ecosystems. Doing so can attain a progressive phasedown and eventual phaseout of chemical insecticides in Asia’s coconut sector.

In recent years, considerable progress has been made integrating semio-chemicals within *O. arenosella* IPM packages. This has involved using pheromone traps to assess BHC seasonal dynamics (Bhanu et al., 2018) or for mass-trapping, i.e., as a direct control measure (Muniyappa et al., 2018). While UV light or kairomone trapping still needs to be further refined, the usage of BHC sex pheromones has been tested and refined in India. A systematic use of (pheromone) traps for monitoring purposes can inform the proper timing and spatial targeting of curative or preventative interventions. Meanwhile, by deploying 40-120 cross-vane traps baited with (ZZZ)-3,6,9-tricosatriene traps per ha, BHC populations are directly reduced. In other assays, a density of 100 pheromone traps per ha resulted in a 34-94% reduction in larval infestation pressure over consecutive generations (Chandrashekharaiah, 2013; Kumara et al., 2015). While these studies underscore the efficacy of semiochemical-based approaches, other scientists have been skeptical about the feasibility or cost-effectiveness of pheromone-based approaches (Fernando and Chandrasiri, 1997; Cork and Hall, 1998). Irrespective of such concerns, one likely can achieve adequate *O. arenosella* control at low trap densities by integrating such measures with augmentative biological control (Kumara et al., 2015).

Similarly as for Tephritid fruit flies (e.g., Deguine et al., 2011), augmentoria can be used to lower BHC population levels and concurrently conserve its resident parasitoid community. These tent-like structures, in which infested palm leaves are deposited, prevent the dispersal of emerged BHC adults while allowing (small-sized) parasitic wasps to recolonize the crop. Those measures, however, remain to be evaluated under different production contexts.

Though Shameer et al. (2018) list multiple intercrop species to enhance parasitoid-mediated BHC control, food web modeling can unveil its underlying mechanics and help to tailor CBC schemes to specific settings (Malard et al., 2020). Given that BHC food webs have only been characterized in Kerala (India), these studies need to be conducted across the *O. arenosella* invaded range. These assays can equally define opportunities to bolster biotic resistance against colonizing invaders in climatically suitable areas, e.g., Indonesia (Levine et al., 2004). In those uninvaded areas, host usage patterns and plant associations of endemic generalists such as *Argyrophylax fumipennis* (Townsend) (Diptera: Tachinidae) also wait to be assessed (Cock and Perera, 1987).

In addition to the above diversification tactics, good agronomic management (e.g., fertilization, irrigation) of standing coconut or other palm crops can boost tree vigor and lower susceptibility to *O. arenosella* attack. More specifically, high dosage of N and P fertilizers increases BHC incidence in coconut, whereas high application rates of potassium decrease pest infestation levels (Sen and Kapadia, 1985). While such approaches may not draw on “cutting edge” science, they still provide feasible, cost-effective, and environmentally-sound management solutions for *O. arenosella* under various conditions.

Endophyte-mediated biological control manages several limiting pests and diseases of perennial crops such as bananas or cacao (Akello et al., 2007). Exploratory work has shown how entomo-pathogenic fungi such as *Beauveria bassiana* (Balsamo) Vuillemin and *Metarhizium anisopliae* (Metsch) endophytically colonize 100% of tissue-culture coconut plantlets (Gaviria et al., 2020). Laboratory assays have also documented the antagonistic effects of various mycorrhizal fungi and bacteria on coconut pests and diseases such as leaf spot disease, basal stem rot disease, or burrowing nematode (Eris et al., 2019). One can build upon this incipient work to investigate whether or not long-term colonization and effective *O. arenosella* control can be achieved with different inoculation techniques, microbiota, and palm species.

Considering how *O. arenosella* has not yet been officially recorded from several Southeast Asian countries (e.g., Indonesia, Philippines, Papua New Guinea), quarantine and biosecurity measures should be implemented to avoid initial pest establishment and subsequent in-country spread. These measures can involve the routine inspection of traded (live) palm plantlets and fresh coconuts from BHC-affected areas. In this regard, much can be learned from programs of China’s Entry-Exit Inspection and Quarantine (CIQ) Department that target various traded agricultural commodities. To detect BHC larvae or cocoons in these substrates, one can sample frass from feeding galleries and collect specimens for further taxonomic identification in the laboratory. The above physical inspections of traded items can be complemented with a whole battery of (molecular, volatile-based) diagnostic tools and the deployment of BHC pheromone traps at or near ports of entry.

Farmers’ deficient agro-ecological knowledge often constitutes a major hurdle in adopting and diffusion biodiversity-based management approaches such as biological control (Wyckhuys et al., 2019). However, participatory research and carefully designed extension programs can fill these knowledge gaps. Farmers receive intensive training through State-supported Community Pest Management Centers in Thailand’s coconut-growing areas. As such, local coconut growers have acquired a sound understanding of *O. arenosella* ecology and have embraced augmentation biological control using *H. hebeto*r (Soontareerat and Treewannakul, 2019). By thus accounting for the social-ecological aspects of BHC pest management, biological control strategies and non-chemical management schemes are being tailored to the diverse agro-ecological and livelihood contexts of tropical Asia.

## Author contributions

HL and JT contributed equally to the work. All authors contributed to the article and approved the submitted version.
